# Uncovering the ecophysiological potential of *Motilimonas* through genomic profiling analysis

**DOI:** 10.1186/s12864-026-12781-0

**Published:** 2026-04-06

**Authors:** Arthur Salengros, Etienne Dechamps, Laurence Meunier, Isabelle F. George

**Affiliations:** Laboratory of Ecology of Aquatic Systems, Brussels School of Bioengineering, Université Libre de Bruxelles (ULB), Brussels, Belgium

**Keywords:** Genome, Phylogeny, Motilimonas, Marine bacteria, Copiotroph, Sugar metabolism, Secretion systems, Chemotaxy, Salt resistance

## Abstract

**Background:**

The *Motilimonas* genus was proposed in 2017 and presently include three recognized species isolated from various environments. This genus is still poorly characterized, and its ability to degrade chitin has recently been reported. A genomic profiling analysis was conducted on the seven *Motilimonas* genomes (family *Psychromonadaceae*) available in the NCBI database.

**Results:**

The phylogenetic study suggests that Motilimonas sp. E26, Motilimonas sp. 1_MG-2023 G1M02 and Motilimonas sp. Spo1_1 could form a new clade distinct from other already existing clades within the Motilimonas genus (i.e. M. cestriensis, M. pumila and M. eburnea). The genomic features of all *Motilimonas* genomes are consistent with a moderately copiotrophic lifestyle. For instance, they encode proteins involved in chemotaxis, motility, type IV pili biosynthesis, sugar phosphotransferase systems (PTS) and chitin degradation. Additional shared traits include aerobic respiration, a preference for sugars over organic acids as carbon sources, the use of a “compatible solute” strategy to tolerate osmotic stress in saline environments, and, except for *M. cestriensis* MKS20^T^, the ability to perform nitrate reduction. Furthermore, all *Motilimonas* genomes encode a diversity of secretion systems. For example, each genome contains one or several complete type I secretion systems (T1SS), one complete T2SS, and four genomes (*Motilimonas* sp. Spo1_1, *M.* sp. E26, *M.* sp. 1_MG-2023 G1M02 and *Motilimonas* sp. KMU-193) harbor a complete type VI secretion system (T6SS). Notably, only *M. pumila* PLHSC7-2^T^ possesses genes encoding a complete type III secretion system (T3SS).

**Conclusions:**

These findings provide new insights into the ecological versatility and adaptive strategies of the *Motilimonas* genus. The next step will involve genome-resolved analyses of metagenomic datasets with the objective to investigate the functional ecology of *Motilimonas* in a broader range of environments contributing to the better understanding of their ecological distribution.

**Graphical Abstract:**

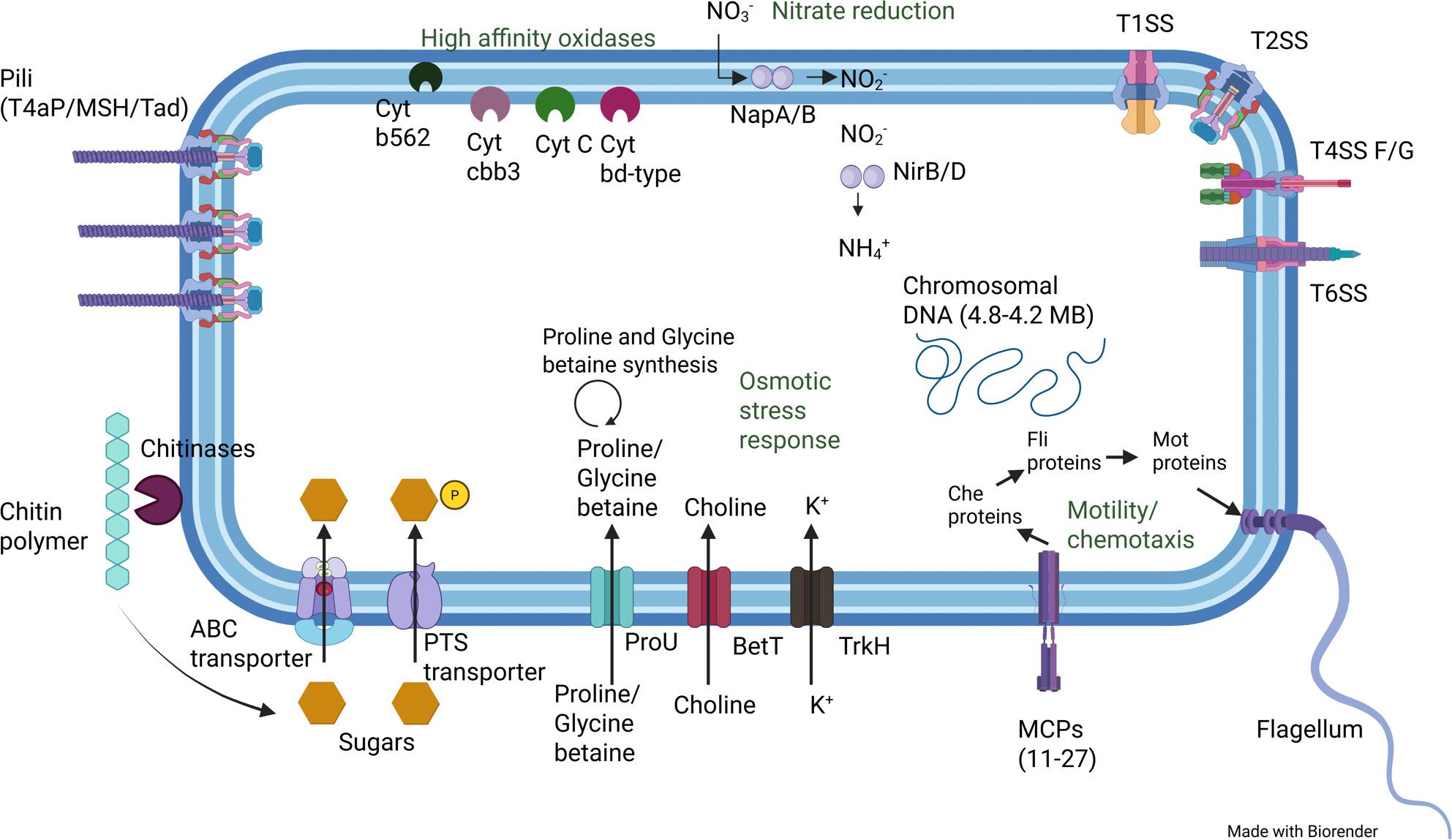

**Supplementary Information:**

The online version contains supplementary material available at 10.1186/s12864-026-12781-0.

## Introduction

In 2017, the genus *Motilimonas* was proposed as a new genus in the *Psychromonadaceae* family, order Enterobacterales, class Gammaproteobacteria based on 16S rRNA gene sequence data as well as physiological and biochemical characteristics by Ling et al. (2017) [1]. It currently comprises three officially recognized species: *M. eburnea* [1], *M. pumila* [2] and *M. cestriensis* [3]. Two potentially novel species were also discovered, one with a suggested name but not validated (*M. chitinivorans*) [4] and one pending validation (*M. thetidivivens*) [5]. Both will be respectively referred as *Motilimonas* sp. Spo1_1 and Motilimonas sp. The strains described were isolated from various environments, either as free-living organisms (in terrestrial brine, marine sediment or seawater) or in association with hosts such as sea cucumbers and sponges. They have been isolated in China, Korea, USA, England and France, suggesting a widespread distribution. The five described *Motilimonas* strains are Gram-negative, heterotrophic, salt-tolerant, facultatively anaerobic bacteria except *Motilimonas* sp. KMU-193. Recently, chitinolytic abilities were demonstrated in *Motilimonas* sp. Spo1_1 using insoluble chitin and colloidal chitin as substrates [4, 6].

In the marine environment, as in other habitats, nutrient concentrations may vary temporally and spatially. The open ocean is generally a nutrient-poor area, while estuarine and coastal waters, including upwelling regions, are nutrient-rich. In this context, the ”oligotroph-copiotroph” concept [7] is used to categorize microorganisms along a continuum of nutrient concentrations. On both endpoints of this spectrum are (i) the oligotrophs that multiply steadily but slowly in nutrient poor environments (as well as in nutrient rich ones) and (ii) the copiotrophs that are able to grow quickly and reproduce in nutrient rich environments but are unable to grow under nutrient limitation, showing a “feast-and-famine” strategy [8–10]. The diversification of trophic strategies along this continuum is reflected in the genome of marine bacteria, as demonstrated by Lauro et al. (2009) [7]. Specific genomic traits can be attributed to a copiotrophic lifestyle. For example, marine copiotrophic bacteria are known to have a larger genome, while oligotrophic ones are more prone to genome streamlining [7, 11]. The genome of copiotrophs harbors a greater number of genes coding for motility and chemotaxis function than that of oligotrophs, indicating the potential need for the former to locate and fully exploit transient nutrient patches [7]. In the copiotrophic lifestyle definition, during periods of starvation, two types of strategies have been revealed by a recent study. Indeed, copiotrophs -termed *limostatic*- shed their flagella and persist in a dormant state until conditions improve while others remain motile, a strategy known as *limokinetic* [12]. Another hallmark of the copiotrophic lifestyle is the presence of numerous phosphotransferase systems (PTS) enabling the uptake of a wide range of sugars. In contrast, oligotrophs generally possess fewer transport systems overall, which are often ATP-binding cassette (ABC) transporters characterized by higher substrate affinity [7, 13].

Finally, copiotrophic microorganisms are often associated with organic particulate matter, such as lake aggregates or marine snow, which are rich in chitin primarily derived from zooplankton [14, 15]. Bacteria that degrade chitin typically attach to the insoluble polymer to enhance its breakdown [16]. Moreover, the presence of chitinase-encoding genes is another characteristic feature of a copiotrophic lifestyle in marine environments [7].

To better understand the ecological strategies and the metabolic potential of the *Motilimonas* species, a genomic profiling analysis of seven publicly available genomes was undertaken. The analyses combined genome annotation, phylogenomics, and functional profiling. In particular, copiotrophic / oligotrophic traits were searched for in order to infer the potential lifestyle of these marine bacteria.

## Materials and methods

### Genomes available

The genome of *Motilimonas* sp. Spo1_1, a strain isolated by our laboratory from the tissue of the intertidal marine sponge *Hymeniacidon perlevis* in 2020 [4, 6], was sequenced in a MinION Mk1C device (Oxford Nanopore Technologies, Oxford, UK) as detailed in Dechamps et al. (2025) [4]. The assembly was performed using FLYE v2.9-b1768 [17] with the –nano-hq parameter. The assembly was visualized with Bandage [18]. K-mer frequency distributions and assembly completeness were assessed using the K-mer Analysis Toolkit (KAT) v2.4.2 with the “comp” parameter [19] and genome completeness was assessed using BUSCO v5.2.2 [20]. This genome is available on NCBI under accession number PRJNA1163305 and assembly number GCA_051055415.1 [4].

All other available *Motilimonas* genomes and associated informations on NCBI were downloaded on the 5th of January 2025: GCA_021295315.1 (*Motilimonas cestriensis* MKS20^T^); GCA_021295345.1 (*Motilimonas eburnea* YH6^T^); GCA_003596335.1 (*Motilimonas pumila* PLHSC7-2^T^); GCA_046452435.1 (*Motilimonas* sp. KMU-193); GCA_021278025.1 (*Motilimonas* sp. E26) and GCA_030547455.1 (*Motilimonas* sp. 1_MG-2023 G1M02). Completeness was checked for those genomes as well using BUSCO v5.2.2.

### Annotation of *Motilimonas* genomes

All genomes were annotated as described in Dechamps et al. (2025) [4]. The genomes were first annotated with Prokka v1.14.6 [21]. The coding sequences identified were then translated into amino acid sequences and functionally annotated against the Cluster of Orthologous Genes (COG) database with eggNOG-mapper [22] Furthermore, each prokka annotation file was functionally annotated against the “genus_prokaryotes” KEGG database using BlastKOALA [21].

In the eggNOG-mapper file, the number of genes coding for proteins belonging to each COG category was counted in each genome. The categories relative to the “eukaryotic” genomes were not taken into account. A new category called “unknown” was created to include the number of genes that had not been assigned to a COG category. The Z-score was calculated for each genome within each COG category using the formula $\frac{x-\mu}{\sigma}$ where *x* represents the number of genes in a given COG category for a specific genome, *µ* is the mean number of genes across all genomes for that COG category, and *σ* is the corresponding standard deviation calculated across all genomes for that COG category. A heatmap including all COG categories was created using GraphPad Prism version 10.1.0 and edited using Inkscape.

### Building phylogenetic trees based on 16S rRNA genes and core genes

To build the 16S phylogenetic tree, four additional genomes from two outgroups were downloaded from NCBI at the same date: GCA_001444405.1 (*Pseudoalteromonas phenolica* KCTC 12086^T^) GCA_900239935.1 (*Pseudoalteromonas carrageenovora* IAM 12662 ATCC43555T^T^), GCA 000420245.1 (*Psychromonas hadalis* ATCC BAA-638^T^) and GCA_000428725.1 (*Psychromonas aquimarina* ATCC BAA-1526^T^).

The 16S rRNA genes were extracted from all genomes using the Bacterial ribosomal RNA predictor v0.9 (Barrnap) (https://github.com/tseemann/barrnap). The top 16S rRNA sense strand (“+”) gene sequence identified by Barrnap for each genome was downloaded, and the seven sequences were grouped in a fasta file. The alignment was done using mafft v7 [23, 24] with the iterative refinement method “E-INS-i” strategy (all other parameters were chosen by default). An unrooted maximum-likelihood phylogenetic tree was computed with 1000 bootstrap repetitions using IQ-TREE v2.3.6 [25]. The selection method ModelFinder Plus [26] was used to find the best parameters by computing the log-likelihoods of an initial parsimony tree for many different models and choosing the model that minimizes the Bayesian information criterion (BIC) score. The best-fit model was TN + F+R2. The tree was viewed and rooted with TreeViewer then edited using Inkscape.

The average nucleotide identity (ANI) value was calculated using the OrthoANIu tool (https://www.ezbiocloud.net/tools/OrthoANIu [27], . The percentage of digital DNA–DNA hybridization (dDDH) between genomes was estimated using the Genome-to-Genome Distance Calculator (GGDC 3.0) with formula 2 [28] .

The analysis of the *Motilimonas* core genome was performed using Roary v3.13.0 [29] with default parameters. Generation of a core alignment using mafft (with the option “-e -- mafft”) was also performed. The core alignment file was extracted from the Roary output. Using the core alignment file, a phylogenetic tree was computed as stated above and the best-fit model was GTR + F+R4. The tree was viewed and rooted with TreeViewer then edited using Inkscape.

### Analysis of genomic features

#### Respiration/ oxygen tolerance

The presence of genes coding for components of the electron chain for oxidative phosphorylation and nitrogen-reducing enzymes was searched for in BlastKOALA files (KEGG pathways). The presence of high-affinity oxidases, according to Morris et Schmidt (2013) [30] and of antioxidant enzymes for neutralizing reactive oxygen species (ROS) were searched for in the eggnog-mapper files (COG categories).

The ability to synthesize the common fermentation end products was searched for in the carbon metabolism and glycolysis KEGG pathways in the BlastKOALA files.

#### Salt resistance strategy

Genes associated with salt resistance were identified using the eggNOG annotations. The genomes were screened for genes involved in the primary response to osmotic stress, i.e. genes related to potassium ion uptake (*kdpF*,* A*,*B*,* C*,*D*,* E* ; *trkG*,* H*,*D*). Genes involved in secondary response (reviewed in [31–33]) were searched for as well. These were the genes coding for choline (*betT*), glycine betaine (*proV*,* W*,*X* also known as *proU*) and carnitine transporters (*caiT*), the non-specific osmoprotectant permease for glycine betaine, proline and ectoine (*proP*), and proteins involved in glycine betain (*betA*,* B*), proline (*proA*,* B*,*C*), ectoine (*ectA*,* B*,*C*), carnitine (*cdhC*,* cdhAB*,* dhcAB*) and trehalose (*tps/tpp*,* treS*,* treY-treZ*,* treP* and *treT*) synthesis.

#### Trophic strategy

The annotated ORFs from 33 specific COG categories across all seven *Motilimonas* genomes were counted according to the copiotrophic/oligotrophic model of Lauro et al. (2009) [7]. Briefly, the authors determined genomic markers of oligotrophic and copiotrophic lifestyles, using respectively the “model” bacteria *Sphingopyxis alaskensis* RB2256 and *Photobacterium angustum* S14. Those markers were then validated using 32 additional genomes coming from the same families (i.e. *Vibrionaceae* or *Sphingomonadaceae*) as well as other known aquatic copiotroph and oligotroph marine bacteria with high or low rRNA operon copy numbers. Counts were expressed relative to the total number of ORFs found in each strain according to prokka v1.14.6 [34]. For each COG category, relative counts were further compared to the reference values defined by Lauro et al. (2009) [7] for an average copiotroph and an average oligotroph. The resulting plot was drawn using ggplot2 (v3.5.2) [35] and edited by Inkscape.

#### Sugar/ acid preferences

All *Motilimonas* genomes were analysed for sugar/acid preferences using the approach described by Gralka et al. (2023) [36]. Briefly, genes involved in acid and sugar metabolism were identified based on the KEGG pathway annotations. This curated gene list was then used to identify and count the corresponding genes in each *Motilimonas* genome. To account for differences in genome size and annotation depth, gene counts were expressed relative to the total number of open reading frames (ORFs) annotated using the eggNOG files. These relative abundances of sugar-related (S) and acid-related (A) genes were then combined using the formula of Gralka et al. (2023) [36] to compute the Sugar-Acid Preference (SAP) index: $SAP=\mathrm{tanh}(sS+aA)$ with s=60.76 and a=-20.21. The SAP index ranges from − 1 to + 1, with values close to − 1 indicating a strong acid-metabolic orientation, and values near + 1 reflecting a strong sugar-metabolic orientation.

#### Transporters

The number of transporters was determined using the BRITE hierarchies implemented in BlastKOALA. In the “Transporters” division of the BRITE hierarchies, the subdivisions “ATP synthase (ATP)-binding cassette (ABC) transporters – saccharide, polyol, and lipid transporters” and “Phosphotransferase system (PTS)” were examined. Within these subdivisions, genes encoding components of transporters were identified and counted. Only transporters presenting the complete set of required machinery components were considered: for ABC transporters, the substrate binding domain (SBP), the transmembrane domains (TMD) and the nucleotide-binding domain (NBD, also known as ATPase) [37]; for PTS transporters, the enzyme I (EI), the phosphocarrier protein HPr and the enzyme II complexes (EII) [38]. The presence of these complete machineries was used as evidence that the bacterium was able to transport the corresponding sugar.

The total number of genes involved in transport in the *Motilimonas* genomes were then compared to 150 other bacterial genomes through the “comparison organisms” feature available on the TransportDB 2.0 website. To account for variations in genome size, the number of genes coding for transporter proteins was expressed relative to genome size (in megabases). Distribution plots were then generated, showing the number of genomes falling within defined intervals of transporter density (e.g., 0–4, 5–10, …, 100–104 transporters per megabase).

#### Sugar polymer degradation

The reference genomes of *Psychromonadaceae* identified in NCBI were downloaded in April 2024 (Table S1). To identify the panel of carbohydrate polymer metabolized by *Motilimonas* spp., and those of the *Psychromonadaceae* family, their genomes were annotated using dbcan3 [39] to extract the genes coding for carbohydrate active enzymes according to Lau & Furusawa (2024) [40].

#### Secretion systems

Secretion systems were identified using TXSScan, a MacSyFinder tool [41, 42]. For genomes assembled at the contig level (see Table 1), the parameter “type of dataset to deal with” was set to “unordered”, whereas for all other genomes it was set to “ordered replicon”. For assemblies at the scaffold level, the parameter “topology of the replicon(s)” was set to “linear”, while for assemblies at the chromosome level, it was set to “circular”. Secretion systems were also identified using the BRITE hierarchies in BlastKOALA. When secretion systems were identified as incomplete as described in Abby et al. (2016) [43], missing genes were manually retrieved whenever possible. Unannotated ORFs within the TXSScan-identified cluster were inspected, and BLASTp [43] was used to identify these ORFs. Plus, up to ten genes located upstream and downstream of the cluster were inspected the same way.

The type IV secretion systems were specifically identified using ConjScan, a MacSyFinder tool [42]. The parameters used were the same as for the TXSScan tool.

#### Chemotaxis and motility

Pili and flagella were identified using TXSScan with the same parameters as those mentioned in the “Secretion systems” section, in conjunction with the BRITE hierarchies implemented in BlastKOALA. When the motility apparatus appeared incomplete according to Abby et al. (2016) [41], missing genes were manually retrieved whenever possible. Unannotated ORFs within the TXSScan-identified cluster were inspected, and BLASTp [43] was used to identify these ORFs. Plus, up to ten genes located upstream and downstream of the cluster were inspected the same way.

Genes coding for methyl-accepting chemotaxis proteins and the aerotaxis/energy sensor Aer were similarly retrieved through searches within the BRITE hierarchies and Pathways of BlastKOALA.

## Results

### Genomic information

**Table 1:** Global genome information

Strains	*Motilimonas* sp. Spo1_1	*Motilimonas* sp. E26	*Motilimonas* sp. 1_MG-2023 G1M02	*Motilimonas* *cestriensis* MKS20^T^	*Motilimonas* *eburnea* YH6^T^	*Motilimonas* sp. KMU-193	*Motilimonas* *pumila* PLHSC7-2^T^
Assembly levels	Chromosome	Scaffold	Contig	Scaffold	Scaffold	Contig	Contig
Sequencing method	Nanopore	Illumina Novaseq	Illumina Nextseq	Illumina Hiseq	Illumina Hiseq	Illumina Hiseq	Illumina Hiseq
Genome contamination (%)	0.47	1.22	1.69	1.65	2.06	1.69	2.15
BUSCO completeness (C) (%)	98.7	98.7	98.7	98.7	98.9	98.8	99.2
Genome size (Mb)	4.8	4.6	4.8	4.8	4.6	4.2	4.5
Nb of contigs	1	84	391	89	71	93	189
G + C ratio (%)	43.8	43.5	43.5	44	46	45.2	45.5
16S rRNA	9	1	2	2	1	1	3
23 S rRNA	9	1	1	1	2	1	3
5 S rRNA	10	2	3	8	6	1	9
tRNA	102	61	87	87	94	91	76
CDS	4257	4181	4290	4325	4137	3751	4047
Sigma factors	6	6	6	6	6	6	6
Transcription factors	69	68	67	72	55	59	56
Habitat	Sponge, Audresselles beach, France (50°49’14.9"N 1° 35’ 34.4"E)	Red macroalgae *Chondrus ocellatus*, China (30°72’N 122°77’E)	Canoe Beach, Nahant, MA, USA (42°25’12.0"N 70°54’25.2"W)	Anderton brine springs, Cheshire, England (53°16’19.7” N 2°31’28.0"W)	Coastal sediment, Weihai, China (36° 54’32.6"N 122°15’16.2"E)	Coastal seawater, Dadaepo area, Busan, Republic of Korea (35°03′12.2’’N 128°57′26.0’’E)	Gut of sea cucumber from Yantai, China (121° 0’13.63"E 37° 44’52.00"N)
Genbank assembly	GCA_051055415.1	GCA_021278025.1	GCA_030547455.1	GCA_021295315.1	GCA_021295345.1	GCA_046452435.1	GCA_003596335.1
Reference	10.1093/jambio/lxaf187	10.1111/1758-2229.13036	10.1101/387191	10.1099/ijsem.0.004763	10.1099/ijsem.0.001621	10.1007/s00284-025-04496-4	10.1099/ijsem.0.003242

All genomes were sequenced with Illumina except *Motilimonas* sp. Spo1_1, which was sequenced with Nanopore technology (Table 1). The assembly levels varied among the genomes analyzed. *Motilimonas* sp. 1_MG-2023 G1M02, *Motilimonas* sp. KMU-193 and *M. pumila* PLHSC7-2^T^ were assembled at the contig level; *Motilimonas* sp. E26, *M. cestriensis* MKS20^T^ and *M. eburnea* YH6^T^ were assembled at the scaffold level while only *Motilimonas* sp. Spo1_1 was assembled as one chromosome with a circular topology. BUSCO completeness scores were high (98.7% to 99.2%) for all assemblies (Table 1). Similarly, the contamination scores were low for all genomes (Table 1).

Genome sizes ranged from 4.2 Mb in *Motilimonas* sp. KMU-193 up to 4.8 Mb in *Motilimonas* sp. Spo1_1, *Motilimonas* sp. 1_MG-2023 G1M02, and *M. cestriensis* MKS20^T^. The number of rRNA genes varied substantially from three copies in *Motilimonas* sp. KMU-193 to 28 in *Motilimonas* sp. Spo1_1. Specifically, *Motilimonas* sp. Spo1_1 contained nine copies of the 16S rRNA gene, *M. pumila* PLHSC7-2^T^ possessed three copies, *Motilimonas* sp. 1_MG-2023 G1M02 and *M. cestriensis* MKS20^T^ had 2 copies each, and the others contained a single copy. The number of sigma factors was identical across all genomes, whereas the number of transcription factors varied from 55 in *M. eburnea* YH6^T^ to 72 in *M. cestriensis* MKS20^T^ (Table 1).

### Phylogenetic insights of the *Motilimonas* genus

**Fig. 1:**
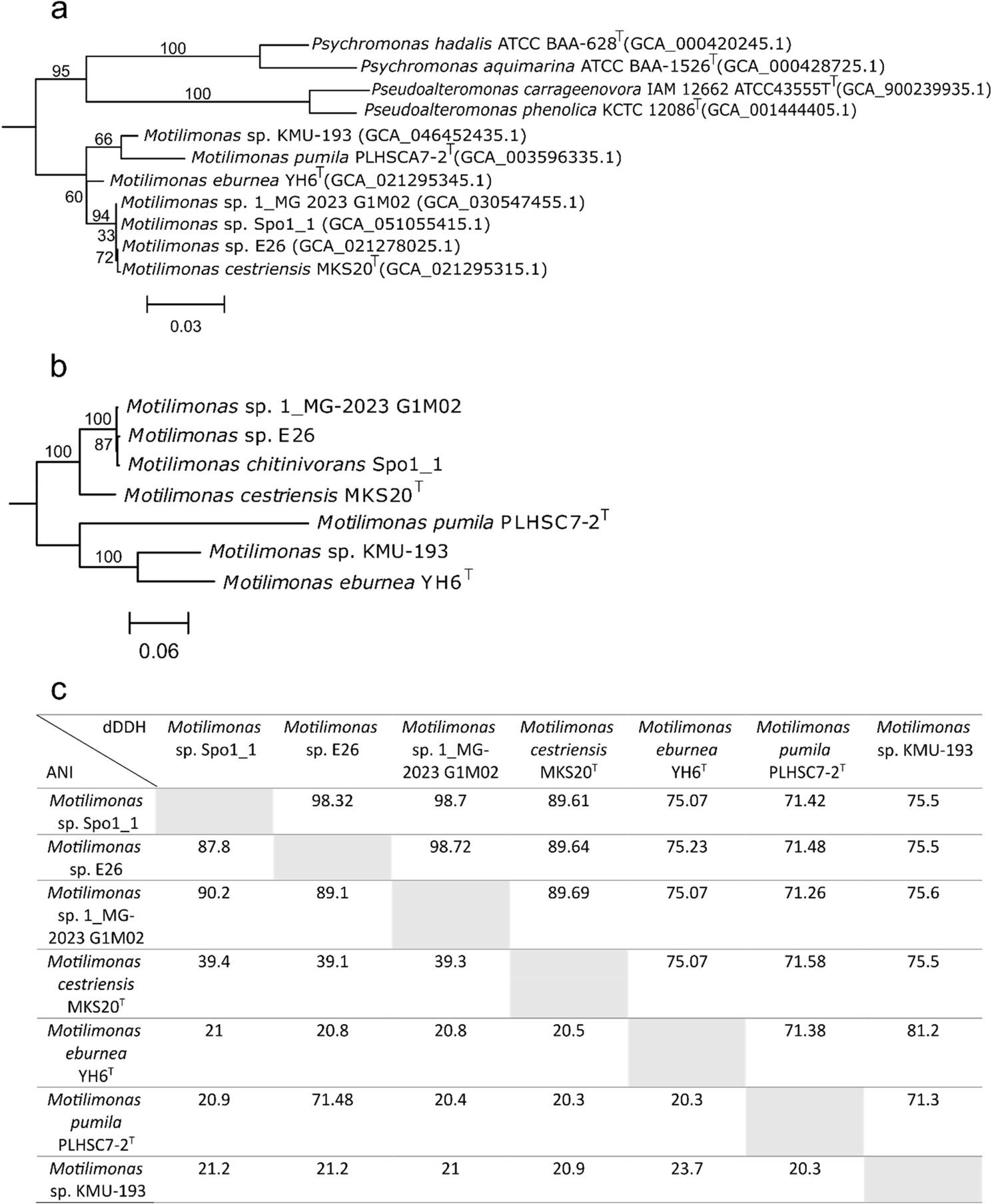
**a** Maximum likelihood phylogenetic tree of the 16S rRNA gene of the Motilimonas genomes, with bootstrap values indicated by nodes. **b** Maximum likelihood phylogenetic tree of the core genome of Motilimonas with bootstrap values indicated by nodes. **c** Average nucleotide identity (ANI) and digital DNA-DNA hybridization (dDDH) comparisons between Motilimonas genomes. Values for ANI are shown on upper right and dDDH on lower left

Based on the 16S rRNA genes, the genus *Motilimonas* is monophyletic, as the two outgroups used (*Psychromonas* and *Pseudoalteromonas*) formed distinct clades. The bootstrap support values for phylogenetic branches within the genus are consistently below 95, indicating that the internal branching of the *Motilimonas* genus is not robust. However, *Motilimonas* sp. 1 MG-2023 G1M02 and sp E26, *Motilimonas* sp. Spo1_1, and *M. cestriensis* MKS20^T^ were more closely related to each other than to the three other strains (Fig. 1a).

To further investigate phylogenetic relationships between *Motilimonas* strains, analysis of OrthoANI, dDDH and the core genome were conducted. *Motilimonas* sp. E26, 1 MG-2023 G1M02 and *Motilimonas* sp. Spo1_1 had OrthoANI values from 98.32% to 98.72% (Fig. 1c). Their dDDH values ranged from 87.8% to 90.2%. Both metrics exceeded the established species threshold by Stackebrandt et al. (2021) of 95% for OrthoANI and 70% for dDDH [44], suggesting a close phylogenetic relationship between these three genomes. In the phylogenetic tree based on core genes, these three genomes formed a distinct clade (Fig. 1b). The closest relative of that clade was *M. cestriensis* MKS20^T^ (Fig. 1b) which showed higher OrthoANI and dDDH values with members of that clade than did the three remaining *Motilimonas* genomes (Fig. 1c). *M. pumila* PLHSC7-2^T^ did not have any OrthoANI and dDDH values above the thresholds with any other *Motilimonas* genome; therefore, it confirmed its status as a distinct species within the *Motilimonas* genus (Fig. 1c). *Motilimonas* sp. KMU-193 and *M. eburnea* YH6^T^ genomes formed a clade and were more closely related to each other than to the other genomes.

### Genomic functional traits

#### General COG categories

Variation in the number of genes assigned to each COG category differed across the *Motilimonas* genomes. Some categories exhibited low variability, with a coefficient of variation (CV) ≤ 5%, including categories C (Energy production and conversion), D (Cell cycle control), and F (Nucleotide transport and metabolism). In contrast, categories A (RNA processing and modification), G (Carbohydrate transport and metabolism), L (Replication and repair), Q (Secondary metabolite biosynthesis), T (Signal transduction), and V (Defense mechanisms) showed higher variability, with CVs ≥ 10%. The remaining categories displayed intermediate variation, with CVs between 5% and 10%.

The closer the genomes phylogenetically (Fig. 1b), the more similar their gene counts per COG category (Fig. S1a and S1b). *Motilimonas* sp. Spo1_1, *Motilimonas* sp. E26, *Motilimonas* sp. 1_MG-2023 G1M02, and *M. cestriensis* MKS20^T^ generally exhibited higher enrichment factors and total gene counts across almost all COG categories compared to the other genomes. (Fig. S1a and S1b). These results can be explained by the larger genome size of those four strains.

The most represented category across all genomes was COG category S (Fig. S1a), corresponding to “Function unknown.” Notably, 17–18% of predicted proteins in each genome fell into that category, highlighting a proportion of genes with uncharacterized function. The created category “unknown” which includes genes for which no information has been found, accounted for 3.8% to 4.6%. These categories may introduce a degree of uncertainty in downstream analyses, but they represent an untapped reservoir of metabolic functionalities to discover.

#### Respiration/ oxygen tolerance

Each genome encoded the complete set of genes required for oxidative phosphorylation. They also harbored multiple antioxidant genes coding for enzymes involved in reactive oxygen species (ROS) detoxification, including several peroxidases and a catalase, except for *Motilimonas* sp. KMU-193, which lacked the catalase gene. All genomes harbored genes coding for high-affinity cytochromes, such as cytochrome cbb3, cytochrome c, cytochrome b562, and cytochrome bd-type (Table 2).

**Table 2:** Oxidative stress defence enzymes and high-affinity cytochromes encoded in the Motilimonas genomes

	Enzymes	COG categories	*Motilimonas* sp. Spo1_1	*Motilimonas* sp. E26	*Motilimonas* sp. 1_MG-2023 G1M02	*Motilimonas* *cestriensis* MKS20^T^	*Motilimonas* *eburnea* YH6^T^	*Motilimonas* sp. KMU-193	*Motilimonas* *pumila* PLHSC7-2^T^
Oxygen tolerance	Catalase	COG0376	1	1	1	1	1	0	1
	Glutathione Peroxidase	COG0386	1	1	1	1	1	1	1
	Peroxidase	COG1858	2	2	2	1	1	1	3
	Peroxidase	COG2128	3	2	2	3	3	2	3
	Peroxidase	COG2837	1	1	1	1	1	1	1
High-affinity cyto chromes	Cytochrome cbb3	COG2010, 4736, 2993 and 3278	1	1	1	1	1	1	1
	Cytochrome bd-type	COG1294 and 1271	1	1	1	1	1	1	1
	Cytochrome b562	COG3783	1	1	1	1	1	1	1
	Cytochrome C oxidase	COG1845, 0843 and 1622	1	1	1	1	1	1	1

According to BlastKOALA annotations, all genomes but that of *M. cestriensis* MKS20^T^ appeared to possess the genetic potential for the reduction of nitrate to ammonia. The genes *napA*, *napB*, *nirB*, and *nirD* were indeed identified in these genomes.

The *Motilimonas* genomes reflected the ability to synthesize diverse common end products of fermentation reactions like lactate, acetate, fumarate, citrate, malate and succinate, suggesting the ability to do fermentation in the absence of oxygen.

#### Salt resistance strategy

All *Motilimonas* genomes analyzed possessed the genes (*proV*, *proX*, *proW*, i.e. *proU*) involved in the transport of proline and glycine betaine into the cell, except for KMU-193 and PLHSC7-2^T^. However, these two strains were the only ones to harbor the genes required for glycine betaine biosynthesis from choline, along with the choline transporter gene (*betT*). Five genomes contained the genes necessary for proline biosynthesis (*proA*, *proB*, and *proC*), as well as two of the three genes (*ectB* and *ectC*) required for ectoine biosynthesis from aspartic acid. The exceptions were *M. pumila* PLHSC7-2^T^, which lacked both *ectB* and *ectC*, and *Motilimonas* sp. E26, which lacked all genes for proline biosynthesis. Overall, each genome encoded at least one system enabling either the transport or the biosynthesis of an osmoprotectant. Furthermore, all genomes harbored several copies of the low-affinity potassium transport system gene (*trkH*).

#### Trophic strategy

**Fig. 2:**
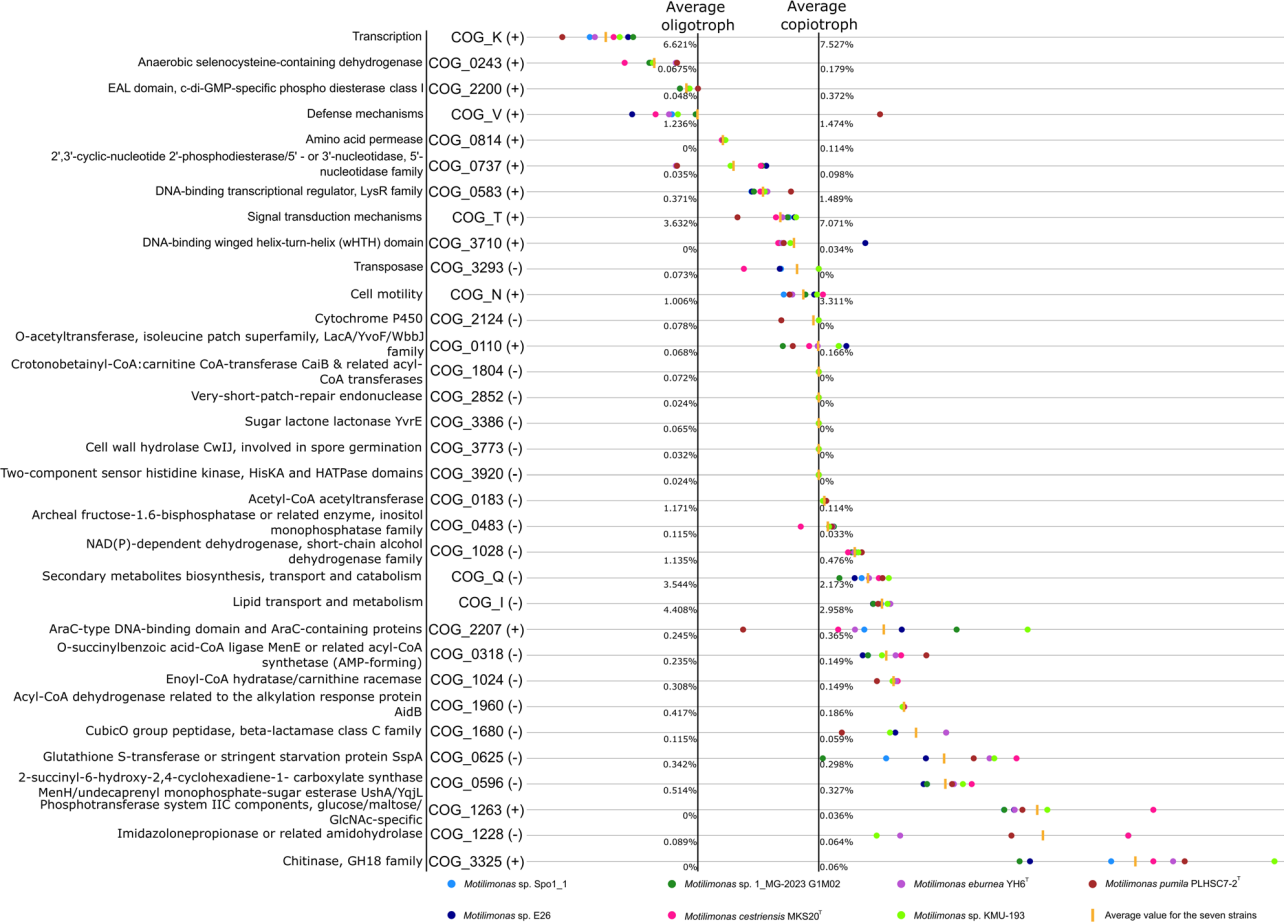
COG categories associated with copiotrophic and oligotrophic lifestyles as defined by Lauro et al. (2009) [7]. Gene counts for each COG category were expressed relative to genome size (in Mb), and subsequently scaled using the copiotroph/oligotroph reference landmarks described by Lauro et al. (2009) [7]. For each COG category, two subcategories are shown: categories in which higher gene counts indicate a more copiotrophic lifestyle (+), and categories in which higher gene counts indicate a more oligotrophic lifestyle (-). Both general and specific COG categories are represented

The trophic strategy of *Motilimonas* strains was studied using a panel of 33 COG categories as described in Lauro et al. (2009) [7]. Fourteen COG categories are categories in which high gene copy numbers were associated with copiotrophy (+ on Fig. 2), whereas for the 19 other categories it is the opposite (- on Fig. 2). Considering the mean percentage for each category across the seven *Motilimonas* genomes, four categories pointed to an oligotrophic lifestyle, eight categories did not allow to discriminate between “average oligotroph” and “average copiotroph”, and 21 pointed to a copiotrophic lifestyle (Fig. 2).

Among these 25 discriminative COG categories, some were general ones and others were subcategories (included in the general ones or not). The general category COG_V (Defense mechanism) classified *Motilimonas* genomes as oligotrophic. However, the three COG_V subcategories —COG_737, COG_2124, and COG_1680— led to a different conclusion: the first two did not allow for discrimination between oligotrophy and copiotrophy, whereas the COG_1680 category classified all genomes as copiotrophs.

The general category COG_K (Transcription) also classified *Motilimonas* genomes as oligotrophic. However, the COG_K subcategories COG_0583 and COG_3710 did not allow for discriminating the *Motilimonas* genome between oligotrophy and copiotrophy.

The general categories COG_T (Signal transduction mechanisms) and COG_N (Cell motility) (Fig. 2) did not allow to infer the trophic strategy of *Motilimonas*.

Finally, the general category COG_Q (Secondary metabolites biosynthesis, transport and catabolism) and COG_I (Lipid transport and metabolism) classified *Motilimonas* as copiotroph bacteria. Consistently, all six COG_I subcategories represented in this analysis (COG_1804, COG_0183, COG_1028, COG_0318, COG_1024, and COG_1960) pointed to a copiotrophic lifestyle as well (Fig. 2).

Among the five most discriminating categories, i.e. those for which the average relative value in *Motilimonas* genomes deviated most from the “average oligotrophic - average copiotrophic” range, were COG_1263, COG_0625 and COG_3325 (Fig. 2). COG_1263 encodes a phosphotransferase system (PTS) subunit IIC involved in the transport of glucose, maltose, and N-acetylglucosamine (GlcNAc). COG_0625 corresponds to either a glutathione S-transferase or the stringent starvation protein SspA. Finally, the most discriminatory category COG_3325 corresponds to chitinase enzymes of the GH18 family. The genomes contained seven genes of that family (in *Motilimonas* sp. 1_MG-2023 G1M02 and *Motilimonas* sp. E26) to eleven genes (in *Motilimonas* sp. KMU-193). Notably, just three genes are sufficient to exceed the “average copiotroph” threshold.

#### Sugar/ acid preferences

The calculated SAP values for the *Motilimonas* genomes ranged from 0.193 (*Motilimonas eburnea* YH6^T^) to 0.731 (*Motilimonas cestriensis* MKS20^T^), with a mean at 0.387. These values indicate that all *Motilimonas* strains had a preference for sugar uptake and degradation compared to acids. These results were consistent with the overrepresentation of sugar transporters (category COG_1263) and chitinase genes (category COG_3325).

#### Transporters

**Fig. 3:**
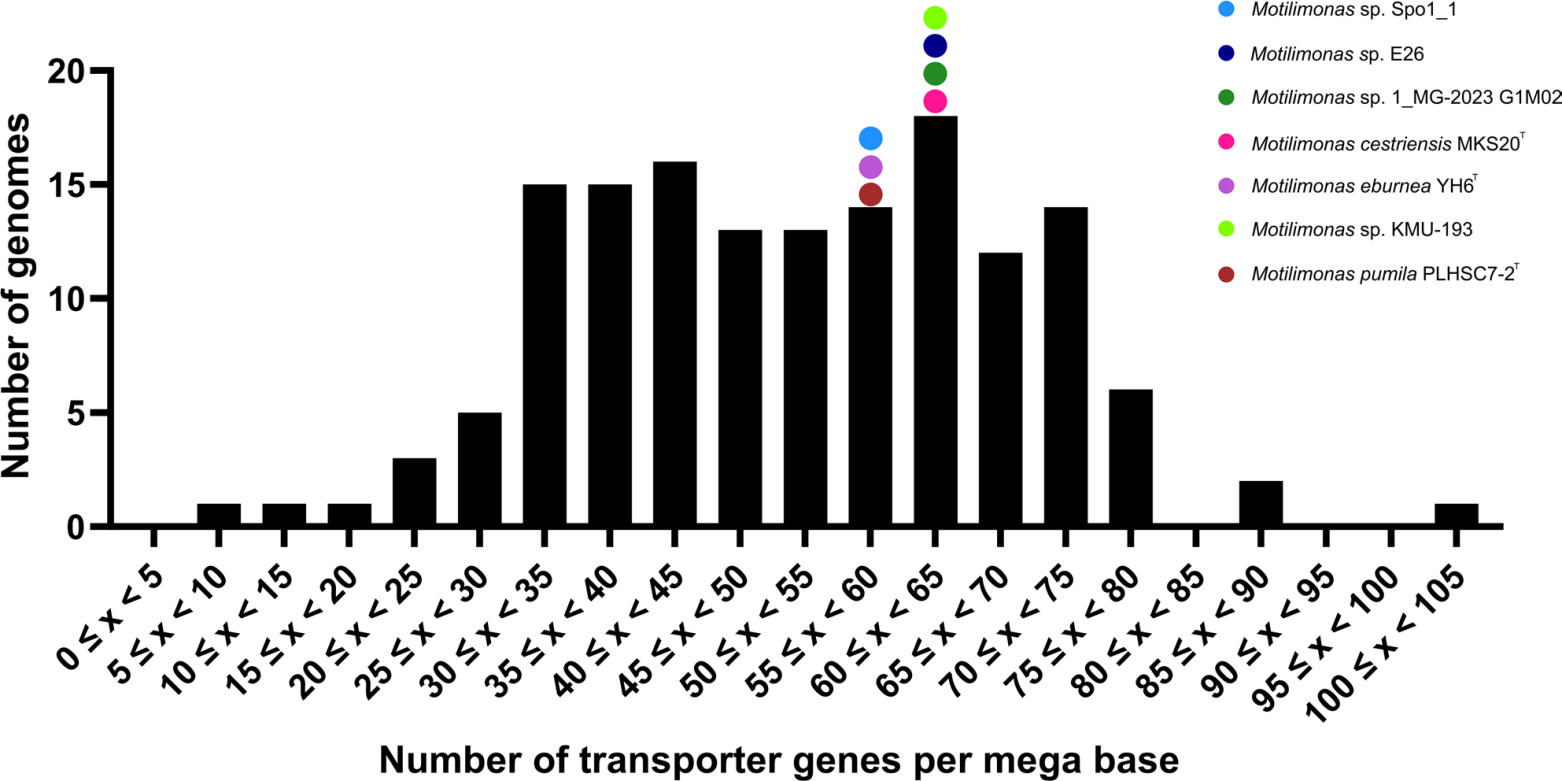
Distribution of genomes according to categories of gene counts involved in the transport of molecules and ions through the cell membrane. 150 bacterial genomes from the TransportDB 2.0 database were used for comparison with the *Motilimonas* genomes. Gene counts were expressed relative to genome size (in Mb). The median number of transporter genes per mega base is 51.39

All *Motilimona*s strains exhibited between 57 and 64 transporter-coding genes per Mb, which is above the median value of 51.4 calculated from 150 bacterial genomes in the TransportDB 2.0 database. This places them within the top 44.7% of genomes with the highest relative transporter gene count (Fig. 3).

*Motilimonas* sp. Spo1_1 and *Motilimonas* sp. E26 possessed a similar total number of transporter genes (280 and 282, respectively), including an identical number of sugar transporter genes [26] (Table 3). These two genomes, along with *Motilimonas* sp. 1_MG-2023 G1M02—which had slightly more sugar transporter genes [32]—displayed comparable normalized transporter gene counts (58.3, 61.3, and 60.2, respectively). All three strains had the capacity to transport the same set of sugars: maltose/maltodextrin, glucose/mannose, ribose, GlcNAc, and fructose. Additionally, *Motilimonas* sp. 1_MG-2023 G1M02 can transport N-acetylgalactosamine.

**Table 3:** Genes involved in the transport of sugars by the two main pathways found in bacteria: ABC and PTS transporters. GlcNAc = N-acetyl-glucosamine, GalNAc = N-acetylgalactosamine

Strain	*Motilimona*s sp. Spo1_1	*Motilimonas* sp. E26	*Motilimona*s sp. 1_MG-2023 G1M02	*Motilimonas* *cestriensis* MKS20^T^	*Motilimonas* *eburnea* YH6^T^	*Motilimonas* sp. KMU-193	*Motilimonas* *pumila* PLHSC7-2^T^
Genome size (Mb)	4.8	4.6	4.8	4.8	4.6	4.2	4.5
Number of transporter genes (per Mb)	280 (58.3)	282 (61.3)	289 (60.2)	294 (61.3)	263 (57.2)	268 (63.8)	256 (56.9)
Sugar transporters							
ABC type genes	16	16	18	21	9	12	9
PTS type genes	11	11	15	20	10	10	10
ABC transported sugars	maltose/ maltodextrine, glucose/mannose, ribose	maltose/ maltodextrine, glucose/ mannose, ribose	maltose/ maltodextrine, glucose/ mannose, ribose	maltose/ maltodextrine, glucose/ mannose, ribose	maltose/ maltodextrine	maltose/ maltodextrine, glucose/mannose	maltose/ maltodextrine
PTS transported sugars	glucose, GlcNAc, fructose	glucose, GlcNAc, fructose	glucose, GlcNAc, fructose, GalNAc	glucose, GlcNAc, maltose/glucose, trehalose, fructose, Mannitol, GalNAc	glucose, GlcNAc	glucose, GlcNAc, fructose	glucose, GlcNAc, mannitol

By contrast, *M. pumila* PLHSC7-2^T^, *M. eburnea* YH6^T^, and *Motilimonas* sp. KMU-193 have lower total numbers of transport-related genes (256, 263, and 268, respectively) and fewer sugar transporters (19, 19, and 22, respectively) (Table 3). While *M. pumila* PLHSC7-2^T^ and *M. eburnea* YH6^T^ have the lowest relative transporter gene counts per Mb (56.9 and 57.2), *Motilimonas* sp. KMU-193 shows the highest (63.8) because of its reduced genome size compared to the other genomes. These three strains are able to transport a narrower range of sugars: maltose/maltodextrin, glucose, and GlcNAc—except for *Motilimonas* sp. KMU-193, which also transports fructose and mannose.

*M*. *cestriensis* MKS20^T^ stands out with the highest total and sugar-specific numbers of transporter genes (294 and 41, respectively) (Table 3). It also exhibits the broadest sugar transport capacity, being able to transport maltose/maltodextrin, glucose/mannose, ribose, GlcNAc, trehalose, fructose, mannitol, and N-acetylgalactosamine.

Notably, all strains are capable of transporting GlcNAc via PTS transporters.

#### Sugar polymer degradation

**Fig. 4:**
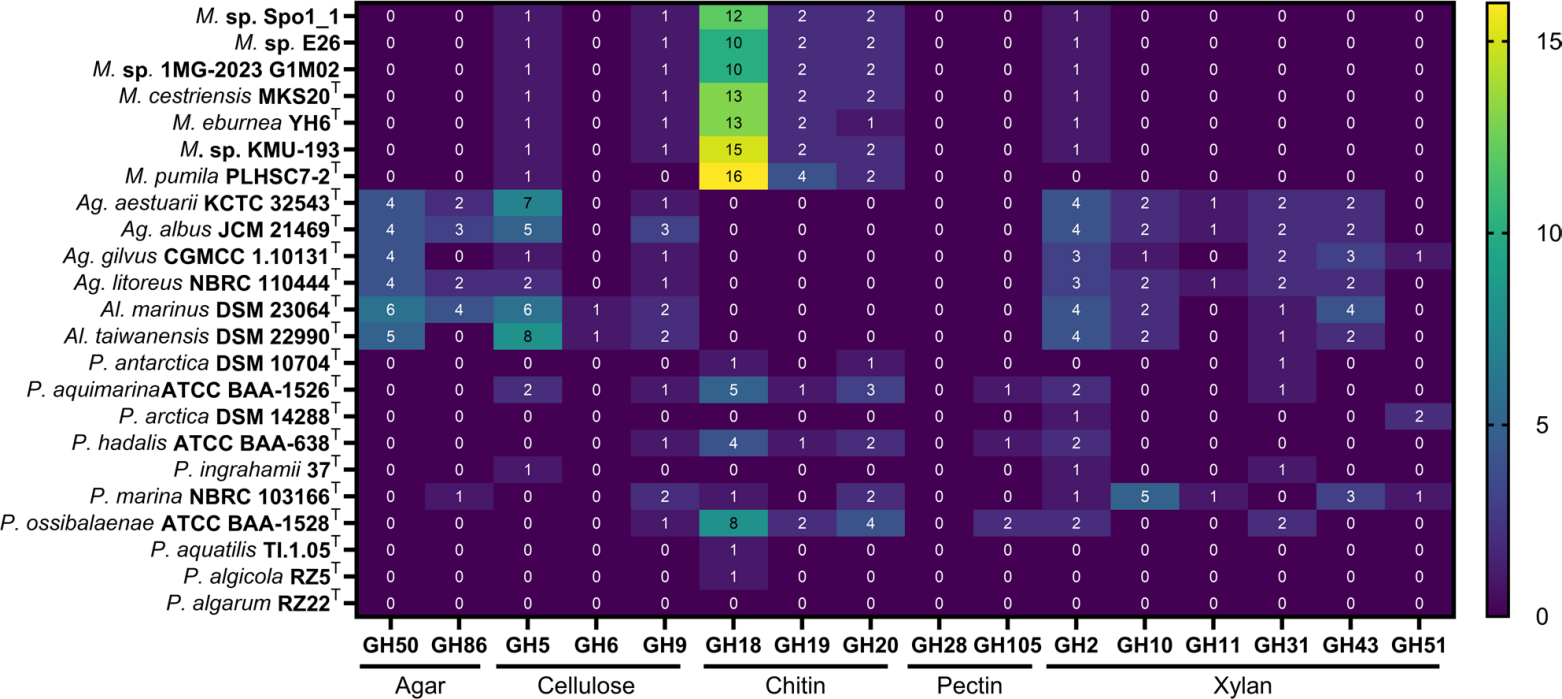
Heatmap of the number of genes coding for glycoside hydrolase (GH) classes involved in agar, cellulose, chitin, pectin and xylan degradation in the genome of *Motilimonas* strains and other *Psychromonadaceae*. Ag = *Agarivorans*, Al = *Aliagarivorans*, P = *Psychromonas*

The *Motilimonas* genomes contained genes encoding all three glycoside hydrolase (GH) families involved in chitin degradation. They possessed between 10 GH18 genes (in *Motilimonas* sp. E26 and *Motilimonas* sp. 1MG-2023 G1M02) and 16 GH18 genes (in *M. pumila* PLHSC7-2^T^) (Fig. 4). All genomes also carried one GH5 gene and one GH2 gene, which are involved in cellulose and xylan degradation, respectively. Aside from these two enzymes, no additional glycoside hydrolases associated with agar, cellulose, pectin, or xylan degradation were identified.

The *Agarivorans* and *Aliagarivorans* genomes contained genes coding for enzymes active in agar (as expected), as well as genes coding for enzymes involved in cellulose (GH5 and GH9 but no GH6) and xylan degradation (Fig. 4).

Five out of seven *Psychromonas* genomes possessed genes coding for enzymes active in chitin degradation, although none harbored as many as the *Motilimonas* genomes. *Psychromonas* genomes exhibited the greatest diversity of glycoside hydrolases for polymer degradation (Fig. 4).

#### Secretion systems

All genomes encoded the complete Tat and Sec clusters (data not shown), as well as at least one copy of the type I secretion system (T1SS) (Table 4). The number of T1SS copies ranged from one in *Motilimonas* sp. 1_MG-2023 G1M02 to three in *Motilimonas* sp. KMU-193, but the majority of *Motilimonas* genomes carried two copies.

**Table 4:** Secretion systems (type one to six) encoded in the *Motilimonas* genomes

Type	Subtype	*Motilimonas* sp. Spo1_1	*Motilimonas* sp. E26	*Motilimonas* sp.1_MG-2023 G1M02	*Motilimonas* *cestriensis* MKS20^T^	*Motilimona*s sp. KMU-193	*Motilimona*s *eburnea* YH6^T^	*Motilimonas* *pumila* PLHSC7-2^T^
T1SS	/	2^a^	2^a^	1^a^	2^a^	1^a^	3^a^	2^a^
T2SS	/	1^a^+1^b^	1^a^+1^b^	1^a^+1^b^	1^a^	1^a^+1^b^	1^a^	1^a^+1^b^
T3SS	/	0	0	0	0	0	0	1^a^
T4SS	T4SS_F	0	0	0	0	0	1^c^	1^d^
T4SS T5SS	T4SS_G	1^d^	0	0	0	0	1^d^	1^d^
	/	0	0	0	0	0	0	0
T6SS	/	1^e^	2^e^	2^e^	0	1^f^	0	0

^a^Complete secretion system detected by TXSScan and confirmed by BlastKOALA (BRITE)

^b^Highly incomplete secretion system detected by TXSScan and BlastKOALA, that was not investigated further using BLASTp

^c^Incomplete type IV secretion system detected by ConjScan but completed using BLASTp (NCBI)

^d^Incomplete type IV secretion system detected by ConjScan

^e^Incomplete secretion system detected by TXSScan and BlastKOALA, but completed using BLASTp (NCBI)

^f^Incomplete secretion system detected by TXSScan, but a complete version was identified using BlastKOALA (BRITE)

Each genome also contained one complete type II secretion system (T2SS), while four genomes harbored one additional, but incomplete, second T2SS clusters: *Motilimonas* sp. E26 (6 out of 13 genes missing), *Motilimonas* sp. 1_MG-2023 G1M02 (7/13 missing), *Motilimonas* sp. KMU-193 (6/13 missing), and *M. pumila* PLHSC7-2^T^ (6/13 missing).

A complete type III secretion system (T3SS) was identified only in *M. pumila* PLHSC7-2^T^.

Three genomes encoded type IV secretion systems (T4SS). *M. eburnea* YH6^T^ and *M. pumila* PLHSC7-2^T^ each possessed both T4SS_F and T4SS_G subtypes, while *Motilimonas* sp. Spo1_1 encoded one T4SS_G. In *M. eburnea* YH6^T^, the T4SS_F cluster was complete, and the T4SS_G cluster was missing four genes: *tfc9*, *tfc17*, *tfc18*, *tfc19* (4/17 missing). In *M. pumila* PLHSC7-2^T^, the T4SS_F cluster was also complete, and the T4SS_G only contained 4 genes out of 17. In *Motilimonas* sp. Spo1_1, three genes (*tfc17*, *tfc18*, *tfc19*) were absent from the T4SS_G cluster.

None of the genomes encoded a type V secretion system (T5SS). In contrast, four genomes—*Motilimonas* sp. Spo1_1, *Motilimonas* sp. E26, *Motilimonas* sp. 1_MG-2023 G1M02, and *Motilimonas* sp. KMU-193—harboured a complete type VI secretion system (T6SS) (Table 4).

#### Chemotaxis and motility

All *Motilimonas* genomes encoded the full set of genes required for flagellar assembly, as well as the complete enzymatic machinery necessary for signal transduction involved in flagellum-mediated motility (Table 5). Indeed, every genome included the genes *cheA*, *cheW*, *cheV*, *cheB*, *cheR*, *cheD*, *cheY*, *cheZ*,* cheX*, *fliG*, *fliM*, *fliN*, *motA* and *mot*B.

**Table 5:** Methyl-accepting chemotaxis protein (MCP) as well as motility systems (flagellum and main type IV filament subcategories (Tad, MSH and T4aP)) encoded in the *Motilimonas* genomes

		*Motilimona*s sp. Spo1_1	*Motilimonas* sp. E26	*Motilimonas* sp. 1_MG-2023 G1M02	*Motilimonas* *cestriensis* MKS20^T^	*Motilimonas* sp. KMU-193	*Motilimonas* *eburnea* YH6^T^	*Motilimonas* *pumila* PLHSC7-2^T^
Chemotaxy	MCP	27	27	25	27	24	21	11
	Aer	1	1	1	1	1	1	0
Motility	Flagellum	1^a^	1^a^	1^a^	1^a^	1^a^	1^a^	1^a^
	Tad	1^b^	0	0	1^b^	0	0	1^c^
	MSH	1^d^	1^d^	1^d^	1^d^	1^d^	1^d^	1^d^
	T4aP	1^e^	1^e^	1^e^	1^e^	1^e^	1^e^	1^e^

^a^Incomplete secretion system detected by TXSScan, but a complete version was identified using BlastKOALA (BRITE)

^b^Incomplete secretion system detected by TXSScan and BlastKOALA, but completed using BLASTp (NCBI)

^c^Highly incomplete secretion system detected by TXSScan and BlastKOALA, that was not investigated further using BLASTp

^d^Complete secretion system detected by TXSScan, but only an incomplete version was identified using BlastKOALA (BRITE)

^e^Complete secretion system detected by TXSScan and confirmed by BlastKOALA (BRITE)

The genomes contained multiple type IV pili systems, either complete or partial. The T4aP and msh subtypes were present and complete in all genomes. Additionally, *Motilimonas* sp. Spo1_1, *M. cestriensis* MKS20^T^, and *M. pumila* PLHSC7-2^T^ harbored complete type IV Tad pili clusters (Table 5).

Regarding chemotaxis, all genomes encoded multiple methyl-accepting chemotaxis proteins (MCPs). The number of MCPs per genome ranged from 11 in *M. pumila* PLHSC7-2^T^ to 27 in *Motilimonas* sp. Spo1_1 and *Motilimonas* sp. E26. None of these MCPs could be functionally annotated, despite additional searches using NCBI BLASTn and InterProScan to complement EggNOG and BlastKOALA predictions. Therefore, it was not possible to determine the specific environmental signals sensed by these MCPs. All genomes, except *M. pumila* PLHSC7-2^T^, presented the MCP involved in aerotaxy (through internal redox sensing) (*aer* gene) (Table 5). No other type of chemoreceptors was identified.

## Discussion

In this study, seven publicly available *Motilimonas* genomes were profiled to elucidate their genomic similarities and differences, with the aim of identifying a conserved set of functional traits characteristic of the genus *Motilimonas*. Six genomes had been sequenced by Illumina and assembled into multiple contigs and scaffolds, and one (Spo1_1) had been sequenced by Nanopore and assembled into one circular chromosome. It is well known that each sequencing technology introduces specific biases inherent to its methodology. With Illumina sequencing, repetitive regions tend to be underrepresented [45], which could explain the variation in the number of 16S rRNA gene copies observed among the *Motilimonas* genomes. For example, Rodriguez et al. (2022) [46] sequenced five *Vagoccoccus fluvialis* genomes with Illumina and Nanopore technologies and showed that assemblies created using only short reads contained less than half of the number of tRNA and rRNA genes found in assemblies created using both long and short reads. The *Motilimonas* genomes completeness obtained from both sequencing approaches was comparable, consistent with observations from previous studies [47]. The raw sequencing data were not available for downloading from NCBI, therefore a reassembly of the genomes could not be performed to reduce assembly-related biases. In the future, the *Motilimonas* strains that are publicly available could be purchased and sequenced using Nanopore technology to assess potential differences.

The size of the *Motilimonas* genomes (4.2 to 4.8 Mb) was superior to the average size of aquatic and host-associated microbial genomes (3.1 and 3.0 Mb, respectively) [48] and close to the median genome size of marine bacteria, i.e. 4.4 Mb [7]. As expected, the *Motilimonas* genomes with the largest sizes exhibited an overrepresentation of COG categories compared with the others.

Based on 16S rRNA gene analysis, four strains were closely related (*Motilimonas* spp. 1 MG-2023 G1M02 and E26, *Motilimonas* sp. Spo1_1, and *M. cestriensis* MKS20^T^) whereas the analysis of their core genes allowed to discriminate two distinct clades in that cluster, the first including Spo1_1, E26, and 1_MG-2023 G1M02 and the second *M. cestriensis* MKS20^T^. This result corroborates previous reports that the 16S rRNA gene is not discriminant enough to infer the phylogeny at the species level [44]. Palmer and colleagues (2020) [49] have shown that a cutoff vamue for a specific set of taxa based on the intraspecies diversity of a group is necessary before performing an ANI analysis. However, such cutoff value does not yet exist for the relatively new taxon *Motilimonas*. All in all, the core genome analysis performed in this study has revealed that the genus *Motilimonas* is currently composed of i) three type strains (*M. pumila*, *M. eburnea* and *M. cestriensis*), *Motilimonas* sp. KMU-193 which is closer to the species *M. eburnea* and whose species name (“*thetidivivens*”) is pending validation [5], and a suggested clade composed of *Motilimonas* sp. Spo1_1, E26 and 1_MG-2023 G1M02.

Whenever possible, genomic characteristics highlighted in this study were compared to phenotypic observations of *Motilimonas* strains described in the literature (Table S2).

All *Motilimonas* genomes contained genes associated with respiratory processes, including those involved in oxidative phosphorylation and reactive oxygen species (ROS) detoxification. This genomic information is consistent with previous descriptions of the physiology of four reference strains, *M. pumila* PLHSC7-2^T^, *M. eburnea* YH6^T^ and *M. cestriensis* MKS20^T^ and *Motilimonas* sp. KMU-193 that were reported to be facultatively anaerobic except for *Motilimonas* sp. which was reported to be obligatory aerobic [1–3, 5]. However, in the reference study of *M. eburnea* YH6^T^, no catalase activity was detected experimentally [1], whereas a gene encoding a catalase enzyme was identified in the corresponding genome. The presence of genes coding for high-affinity oxidases (enabling the harvest of O_2_ at nanomolar concentrations) in all genomes suggests that *Motilimonas* spp. are microaerobic, as defined by Morris & Schmidt (2013) [30]. Furthermore, the presence or absence of genes involved in nitrate reduction pathways aligns with experimental observations from the reference studies: *M. pumila* PLHSC7-2^T^ and *M. eburnea* YH6^T^ are able to reduce nitrate, whereas *M. cestriensis* MKS20^T^ lacks this capacity [1–3].

The seven genomes displayed adaptive traits to marine environments consistent with the “compatible solute” strategy. In contrast to the “salt-in” strategy, which is restricted to organisms inhabiting hypersaline environments (e.g., *Halobacterium salinarum* NRC-1, capable of growth at 15–30% [w/v] NaCl) [50], the “compatible solute” strategy is the predominant mechanism of osmoadaptation in bacteria and especially in marine bacteria [31]. This strategy does not require major genetic modifications and confers high flexibility to cope with fluctuating environmental conditions [31, 33]. The observed growth ranges of *Motilimonas pumila* PLHSC7 (1–8% NaCl [w/v]), *Motilimonas eburnea* YH6^T^ (1–9.5%), *Motilimonas* sp. KMU-193 (0–3%) and *Motilimonas cestriensis* MKS20^T^ (0.5–10%) are consistent with this strategy [1–3, 5].

We suggest to classify *Motilimonas* spp. as “moderate copiotrophs”, as they were assigned to the ‘copiotrophic’ strategy for 21 of the 33 COG (sub)categories proposed by Lauro et al. (2009) [7] to differentiate oligotrophs and copiotrophs. Among those, the COG category 0625 (“Glutathione S-transferase or stringent starvation protein SspA”) includes genes involved in the stringent response. The SspA protein, which shares homology with glutathione S-transferases, has been shown to interact with (p)ppGpp, a molecule that accumulates at high concentrations in cells under nutrient starvation [51]. This aligns with the feast-and-famine dynamics that characterize a copiotrophic lifestyle [7, 13]. Motility is another key feature of copiotrophs, which rely on locating and migrating towards nutrient-rich patches [7]. This trait is represented by the COG_N category; yet, based on our analysis, it remains unclear whether it can be used to clearly classify the *Motilimonas* genomes as oligotrophic or copiotrophic. However, consistent with a copiotrophic lifestyle, *Motilimonas* genomes encode the complete flagellar biosynthesis machinery, as well as multiple type IV pili (T4P) systems, including MSH, T4aP, and Tad. Type IV pili are essential for the early stages of particle colonization, as they mediate adhesion to surfaces [52]. The various T4P subtypes share overlapping functions, including DNA uptake, biofilm formation, twitching motility, and substrate adherence [53]. Among these, MSH and Tad remain comparatively less studied than T4aP [53, 54]. In *Vibrio cholerae*, MSH pili were shown to be involved in braking and anchoring within viscous environments, and to be associated with virulence [55]. The Tad pilus, typically encoded within a single genetic locus, has been shown to frequently be lost or acquired, resulting in an uneven distribution even among isolates of the same species [54]. This variability might explain why Tad loci were only found in *Motilimonas* sp. Spo1_1, *M. cestriensis* MKS20^T^, and *M. pumila* PLHSC7-2^T^. The presence of T4P systems might be consistent with the ecological role of *Motilimonas.* Indeed, *Motilimonas* sp. Spo1_1 was demonstrated to be an efficient degrader of insoluble chitin [4], and chitin is largely encountered in particulate form in marine environments [56]. Furthermore, this metabolic ability is encoded in the genome of the seven *Motilimonas* strains (see here below).

In addition to motility, copiotrophic bacteria must be able to locate nutrient patches. This process relies on sensory and signaling systems that modulate flagellar rotation, thereby biasing swimming behavior toward longer forward runs and fewer tumbles—i.e., chemotaxis [57]. Methyl-accepting chemotaxis proteins (MCPs) are the predominant chemoreceptors in bacteria; they act as sensors in two-component signaling systems [57, 58]. The *Motilimonas* genomes were found to contain between 11 and 27 putative MCPs, a number that is considerably lower than the 45 identified in *Vibrio cholerae* [59] but higher than the average 13.9 MCPs per genome [60] except for *M. pumila* PLHSC7-2^T^. MCPs are also involved in different cellular activities like biofilm formation, flagellum biosynthesis, or degradation of xenobiotic compounds [58]. The fact that we could not functionally annotate these MCPs reflects biases in chemotaxis research, which is still largely based on a few model organisms such as *Escherichia coli* and *Bacillus subtilis* [61]. Among the *Motilimonas* putative MCPs, only the Aer-type receptor— mediating aerotaxis (indirectly through energy-sensing mechanisms that detect shifts in the cell’s internal redox balance) [62, 63] —was detected in six out of the seven genomes.

All *Motilimonas* genomes reflected their ability to degrade chitin. This is illustrated by the presence of genes classified under COG_3325 (extracellular GH18 chitinases) and COG_1263 (PTS IIC components specific for glucose/maltose/GlcNAc). Both categories were abundant across all genomes and are considered signatures of pronounced copiotrophy by Lauro et al. (2009) [7]. It is, for example, well established that *Vibrio* spp. are both copiotrophic [13] and efficient chitin colonizers [56] and degraders [64, 65]. The overrepresentation of chitinase genes relative to those coding for other glycosyl hydrolase classes in *Motilimonas* genomes and the presence of complete ABC or PTS transport systems for maltose/maltodextrin, glucose, and GlcNAc (the chitin monomer) indicated a specialization of *Motilimonas* in chitin degradation compared with other genera within the *Psychromonadaceae*. This finding backs up experimental evidence showing that Spo1_1 is an efficient chitin degrader [4].

According to the SAP model [36], all *Motilimonas* genomes reflected a general preference for sugar metabolism. Consistent with the presence of the associated transporters identified in this study, experimental assays showed that *M. cestriensis* MKS20^T^ can oxidize maltose, glucose, mannose, GlcNAc, trehalose, fructose, mannitol, and N-acetylgalactosamine [3]. The same can be said for *M. pumila* PLHSC7-2^T^, which is able to oxidize maltose, glucose, and GlcNAc [2], and for *M. eburnea* YH6^T^ which can oxidize glucose and GlcNAc [1]. Both strains, however, were also able to oxidize mannose, fructose, and N-acetylgalactosamine [1, 2], despite lacking complete transport systems for these sugars in their genome. It is possible that these sugars are taken up via non-specific transporters instead, namely the “multiple sugar transport system ATP-binding proteins” K10112 (*msmX*,* msmK*,* malK*,* sugC*) and K10111 (*malK*,* mtlK*,* thuK*), which are present in both genomes. Finally, genome analysis has revealed that *M. eburnea* can transport maltose and *M. pumila* can transport mannitol, although the oxidation of these compounds has not been experimentally demonstrated by these two strains, respectively [1, 2]. A parallel can be drawn between the phylogenetic relatedness of the *Motilimonas* strains and their sugar transport capacities. Specifically, the clade containing *Motilimonas* sp. Spo1_1, *Motilimonas* sp. E26, and *Motilimonas* sp. 1_MG-2023 G1M02 encodes the second highest diversity and number of sugar transporters, surpassed only by its closest phylogenetic neighbor, *M. cestriensis* MKS20^T^. Among these transporter genes, the presence of PTS transporter genes indicates a copiotrophic lifestyle, as oligotrophs typically possess fewer or no PTS transporter(s) and have a relatively higher number of genes encoding high-affinity ABC transporters [13]. The fact that *Motilimonas* genomes encoded more transporter genes per Mb than the median (51.4%) of the 150 genomes in TransportDB 2.0 provides additional evidence for their association with a copiotrophic lifestyle [7, 13].

After considering genomic traits related to motility, chemotaxis, chitin degradation, and sugar transport, we analyzed secretion systems in *Motilimonas* genomes. The number and diversity of secretion systems identified appear consistent with the observation that Gammaproteobacteria is the second-most represented phylum in terms of proportion of genomes encoding at least one system in each category (T1SS, T2SS, T3SS, T4SS, T5SS, T6SS) [41]. The *Motilimonas* genomes harbored between one and two clusters encoding T1SS, consistent with the observation that T1SS is among the most widely represented secretion systems in diderms; indeed, more than half of the 128 genomes of bacteria with LPS-containing outer membrane analyzed by Abby et al. (2016) [41] contained at least one T1SS cluster (821/1528). The type I secretion system (T1SS) is best known for its role in toxin secretion, notably hemolysin A in uropathogenic *Escherichia coli*. However, T1SS can also secrete a variety of other unfolded proteins, including proteases, lipases, and adhesins [66, 67]. This raises questions regarding the role of the T1SS in *Motilimonas spp*.

In contrast to T1SS, T2SS is less common among diderms, with only 360 out of 1528 genomes containing at least one T2SS cluster [41]. Moreover, T2SS was detected in genomes from only 32 genera within the Pseudomonadota phylum [68], making the presence of at least one cluster in each *Motilimonas* genome unexpected. Additionally, the incomplete T2SS observed in four of the seven genomes can be explained by the frequent horizontal transfer of complete or partial T2SS clusters, which often result in incomplete and nonfunctional systems [41]. Similarly to T1SS, the type II secretion system (T2SS) is classically associated with toxin secretion in pathogenic bacteria, such as the cholera toxin. Nevertheless, T2SS is also widely used by environmental bacteria to export diverse hydrolytic enzymes, including chitinases. Chitinase secretion via T2SS has been documented in *Vibrio cholerae*, *Burkholderia pseudomallei*, *Vibrio vulnificus*, and *Legionella pneumophila* [68, 69]. The presence of T2SS-encoding genes across all *Motilimonas* genomes further supports the hypothesis that chitinolytic capabilities are conserved at the genus level. Moreover, it is consistent with the presence of Sec-dependent signal peptides identified in all endochitinase sequences of *Motilimonas* sp. Spo1_1 by Dechamps et al. (2025) [4], as Sec translocation often precedes secretion through T2SS [68].

Due to its structural and functional properties, the type III secretion system (T3SS) is commonly used for the direct translocation of effector proteins into prokaryotic or eukaryotic cells. Pathogens such as *Salmonella* employ T3SS to deliver effectors into host cells during invasion [66, 70], while symbiotic bacteria such as *Rhizobium* use it to secrete proteins essential for plant symbiosis [70, 71]. Among *Motilimonas* isolates, only *M. pumila* PLHSC7-2^T^ and *Motilimonas* sp. Spo1_1 were isolated from eukaryotic hosts (the gut of a sea cucumber and the tissue of a sponge, respectively) [2, 4]. The presence of a complete T3SS in *M. pumila* PLHSC7-2 ^T^ is therefore noteworthy, as it may enable secretion of effectors that promote symbiosis with the host or, alternatively, reflect a pathogenic interaction.

In addition, several complete or partial type IV secretion systems (T4SS) were identified in the *Motilimonas* genomes, all chromosomally encoded since no plasmids were detected. Two subtypes were present among the eight known T4SS classes: T4SS_F and T4SS_G [42]. In *Motilimonas*, T4SS_G appeared either incomplete (*Motilimonas* sp. Spo1_1 and *M. eburnea* YH6^T^) or degenerated (*M. pumila* PLHSC7-2^T^). When complete, these systems are located within genomic islands, of which they mediate the transfer by conjugation [72]. Their occurrence likely reflects past genomic island insertions, which are known to introduce traits related to pathogenicity, antimicrobial resistance, or symbiosis in the chromosome [73]. This reinforces the potential for either symbiotic or pathogenic interactions of *M. pumila* PLHSC7-2^T^ with its eukaryotic host. In addition, a complete T4SS_F was identified in *M. eburnea* YH6^T^ and *M. pumila* PLHSC7-2^T^. Although T4SS_F are most commonly found on plasmids, plasmids can become integrative and conjugative elements (ICEs) through the acquisition of an integrase, while conversely, ICEs may evolve into plasmids if they acquire a replication system [74, 75]. This bidirectional transition could explain the chromosomal occurrence of T4SS_F in both genomes. The function of these chromosomally encoded T4SS_F remains uncertain, but they may facilitate the horizontal transfer of ICEs [74, 75].

Surprisingly, no T5SS was detected in *Motilimonas* genomes, despite this system (T5SSa, b, or c) being encoded in 62% of diderm genomes according to Abby et al. (2016). In contrast, T6SS were identified in the genomes of *Motilimonas* sp. Spo1_1, *Motilimonas* sp. E26, *Motilimonas* sp. 1_MG-2023 G1M02, and *Motilimonas* sp. KMU-193. The presence of these systems may represent adaptations to ecological pressures, enabling the delivery of cytotoxic effectors into either bacterial competitors or eukaryotic target cells, thereby supporting both ecological niche maintenance and potential host invasion [66, 76].

## Conclusion

This genomic profiling study provides the first comprehensive overview of the metabolic, ecological, and taxonomic features of the *Motilimonas* genus. However, a limitation of this work is the scarcity of publicly available genomes, which likely do not fully capture the genomic diversity within this genus. The next step will involve genome-resolved analyses of metagenomic datasets, either publicly available or generated in our laboratory, with the aim of investigating the functional diversity of *Motilimonas* in a wider range of environments. These analyses will contribute to a more comprehensive assessment of their ecological distribution, functional expression, and contributions to biogeochemical processes at a broader and more representative scale.

## Supplementary Information

Supplementary Material 1.

## Data Availability

The genome of *Motilimonas* sp. Spo1_1 is available on NCBI under accession number PRJNA1163305 and assembly number GCA_051055415.1. All other available *Motilimonas* genomes on NCBI were downloaded on the 5th of January 2025: GCA_021295315.1 ( *Motilimonas cestriensis* MKS20); GCA_021295345.1 ( *Motilimonas eburnea* YH6); GCA_003596335.1 ( *Motilimonas* pumila PLHSC7-2); GCA_046452435.1 ( *Motilimonas* sp. KMU-193); GCA_021278025.1 ( *Motilimonas* sp. E26) and GCA_030547455.1 ( *Motilimonas* sp. 1_MG-2023 G1M02).Additionnal genomes were downloaded for other genomic and phylogenetic analyses: GCA_001444405.1 ( *Pseudoalteromonas phenolica* KCTC 12086) GCA_900239935.1 ( *Pseudoalteromonas carrageenovora* IAM 12662 ATCC43555T); GCA 000420245.1 ( *Psychromonas hadali*s ATCC BAA-638); GCA_000428725.1 ( *Psychromonas aquimarina* ATCC BAA-1526); GCA_022267535.1 ( *Psychromonas antarctica* DSM 10704); GCA_000428725.1 ( *Psychromonas aquimarina* ATCC BAA-1526); GCA_000482725.1 ( *Psychromonas arctica* DSM 14288); GCA_000420245.1 ( *Psychromonas hadalis* ATCC BAA-638); GCA_000015285.1 ( *Psychromonas ingrahamii* 37); GCA_030161115.1 ( *Psychromonas marina* NBRC 103166); GCA_000381745.1 ( *Psychromonas ossibalaenae* ATCC BAA-1528) GCA_019670125.1 ( *Agarivorans aestuarii* KCTC 32543); GCA_019670105.1 ( *Agarivorans albus* JCM 21469); GCA_014636215.1 ( *Agarivorans gilvus* CGMCC 1.10131); GCA_019649015.1 ( *Agarivorans litoreus* NBRC 110444); GCA_000429485.1 ( *Aliagarivorans marinus* DSM 23064) and GCA_000429505.1 ( *Aliagarivorans taiwanensis* DSM 22990).
