# Supplementary material for: Uncovering the ecophysiological potential of Motilimonas through genomic profiling analysis
Source: BMC Genomics. 2026 Apr 6;27:470. doi: 10.1186/s12864-026-12781-0 (PMC13182010; doi:10.1186/s12864-026-12781-0)
Supplement: Supplementary file 1 — Supplementary Material 1. [file 12864_2026_12781_MOESM1_ESM.docx]

**Supplementary Figures & Tables**

Table S1: GenBank reference of the different *Psychromonadaceae* genomes included in Figure 4.

| Strain | GenBank reference |
| --- | --- |
| *Agarivorans aestuarii* KCTC 32543^T^ | GCA_019670125.1 |
| *Agarivorans albus* JCM 21469^T^ | GCA_019670105.1 |
| *Agarivorans gilvus* CGMCC 1.10131^T^ | GCA_014636215.1 |
| *Agarivorans litoreus* NBRC 110444^T^ | GCA_019649015.1 |
| *Aliagarivorans marinus* DSM 23064^T^ | GCA_000429485.1 |
| *Aliagarivorans taiwanensis* DSM 22990^T^ | GCA_000429505.1 |
| *Psychromonas antarctica* DSM 10704^T^ | GCA_022267535.1 |
| *Psychromonas aquimarina* ATCC BAA-1526^T^ | GCA_000428725.1 |
| *Psychromonas arctica* DSM 14288^T^ | GCA_000482725.1 |
| *Psychromonas hadalis* ATCC BAA-638^T^ | GCA_000420245.1 |
| *Psychromonas ingrahamii* 37^T^ | GCA_000015285.1 |
| *Psychromonas marina* NBRC 103166^T^ | GCA_030161115.1 |
| *Psychromonas ossibalaenae* ATCC BAA-1528^T^ | GCA_000381745.1 |

Table S2: Comparison between the identified genomic characteristics and the observed phenotype of the *Motilimonas* strains. *Phenotypic information was retrieved from [1-3] and [5].* / = not tested , GlcNAc = N-acetyl-glucosamine, GalNAc = N-acetylgalactosamine

|  | Strain | *Motilimonas* sp. Spo1_1 | *Motilimona*s sp. E26 | *Motilimonas*sp. 1_MG-2023 G1M02 | *Motilimonas*  *cestriensis* MKS20^T^ | *Motilimonas*  *eburnea* YH6^T^ | *Motilimonas*  sp.KMU-193 | *Motilimonas*  *pumila*  PLHSC7-2^T^ |
| --- | --- | --- | --- | --- | --- | --- | --- | --- |
| Salt resistance strategy | Genomic traits | Proline & glycine betaine transport (*proU*), proline biosynthesis (*proA,B,C*) and ectoine biosynthesis (*ectB &* ectC).  Potassium transport (*trkH*) | Proline & glycine betaine transport (*proU*) and ectoine biosynthesis (*ectB &* ectC).  Potassium transport (*trkH*) | Proline & glycine betaine transport (*proU*), proline biosynthesis (*proA,B,C*) and ectoine biosynthesis (*ectB &* ectC).  Potassium transport (*trkH*) | Proline & glycine betaine transport (*proU*), proline biosynthesis (*proA,B,C*) and ectoine biosynthesis (*ectB &* ectC).  Potassium transport (*trkH*) | Proline & glycine betaine transport (*proU*), proline biosynthesis (*proA,B,C*) and ectoine biosynthesis (*ectB &* ectC).  Potassium transport (*trkH*) | Proline biosynthesis (*proA,B,C*) and ectoine biosynthesis (*ectB &* ectC).  Potassium transport (*trkH*) | Proline biosynthesis (*proA,B,C*)  Potassium transport (*trkH*) |
|  | Phenotype | / | / | / | NaCl optimal growth : 1-3% (w/v) | NaCl optimal growth : 2-4% (w/v) | NaCl optimal growth : 1% (w/v) | NaCl optimal growth : 2-3% (w/v) |
| Sugar metabolism | Genomic traits | Complete gene set for transport of:  maltose/ maltodextrine, glucose/mannose,  ribose, GlcNAc, fructose | Complete gene set for transport of:  maltose/ maltodextrine, glucose/mannose,  ribose, GlcNAc, fructose | Complete gene set for transport of: maltose/ maltodextrine, glucose/mannose,  ribose, GlcNAc, fructose, GalNAc | Complete gene set for transport of:  maltose/ maltodextrine, glucose/mannose, ribose, GlcNAc, trehalose, fructose, Mannitol, GalNAc | Complete gene set for transport of:  maltose/ maltodextrine, glucose, GlcNAc | Complete gene set for transport of:  maltose/ maltodextrine, glucose/mannose, GlcNAc, fructose | Complete gene set for transport of: maltose/ maltodextrine, glucose, GlcNAc, mannitol |
|  | Phenotype | / | / | / | Oxidation of :  maltose, mannitol, GalNAc, glucose, mannose, GlcNAc | Oxidation of :  fructose, GalNAc, glucose, mannose, GlcNAc | Oxidation of :  fructose, glucose, mannose, GlcNAc | Oxidation of :  maltose, GalNAc, glucose, mannose, GlcNAc |
| Sugar polymer degradation | Genomic traits | No genes for pectin or agar degradation  Genes for chitin degradation | No genes for pectin or agar degradation  Genes for chitin degradation | No genes for pectin or agar degradation  Genes for chitin degradation | No genes for pectin or agar degradation  Genes for chitin degradation | No genes for pectin or agar degradation  Genes for chitin degradation | No genes for pectin or agar degradation  Genes for chitin degradation | No genes for pectin or agar degradation  Genes for chitin degradation |
|  | Phenotype | Chitin (+) | / | / | Pectin (-) | Pectin (-) | Pectin (-)  Agar (-) | Pectin (-) |
| Motility | Genomics traits | Complete set of genes for motility | Complete set of genes for motility | Complete set of genes for motility | Complete set of genes for motility | Complete set of genes for motility | Complete set of genes for motility | Complete set of genes for motility |
|  | Phenotype | / | / | / | Motile | Motile | Motile | Motile |
| Oxygen requirement | Genomic traits | High-affinity oxidases  ROS detoxification | High-affinity oxidases  ROS detoxification | High-affinity oxidases  ROS detoxification | High-affinity oxidases  ROS detoxification | High-affinity oxidases  ROS detoxification | High-affinity oxidases  ROS detoxification | High-affinity oxidases  ROS detoxification |
|  | Phenotype | / | / | / | Facultatively anaerobic | Facultatively anaerobic | Obligately aerobic | Facultatively anaerobic |
| Nitrate reduction | Genomic traits | *napA, napB, nirB, nirD* (genes for nitrate reduction) | *napA, napB, nirB, nirD* (genes for nitrate reduction) | *napA, napB, nirB, nirD* (genes for nitrate reduction) | No gene | *napA, napB, nirB, nirD* (genes for nitrate reduction) | *napA, napB, nirB, nirD* (genes for nitrate reduction) | *napA, napB, nirB, nirD* (genes for nitrate reduction) |
|  | Phenotype | / | / | / | Nitrate reduction (-) | Nitrate reduction (+) | Nitrate reduction (+) | Nitrate reduction (+) |


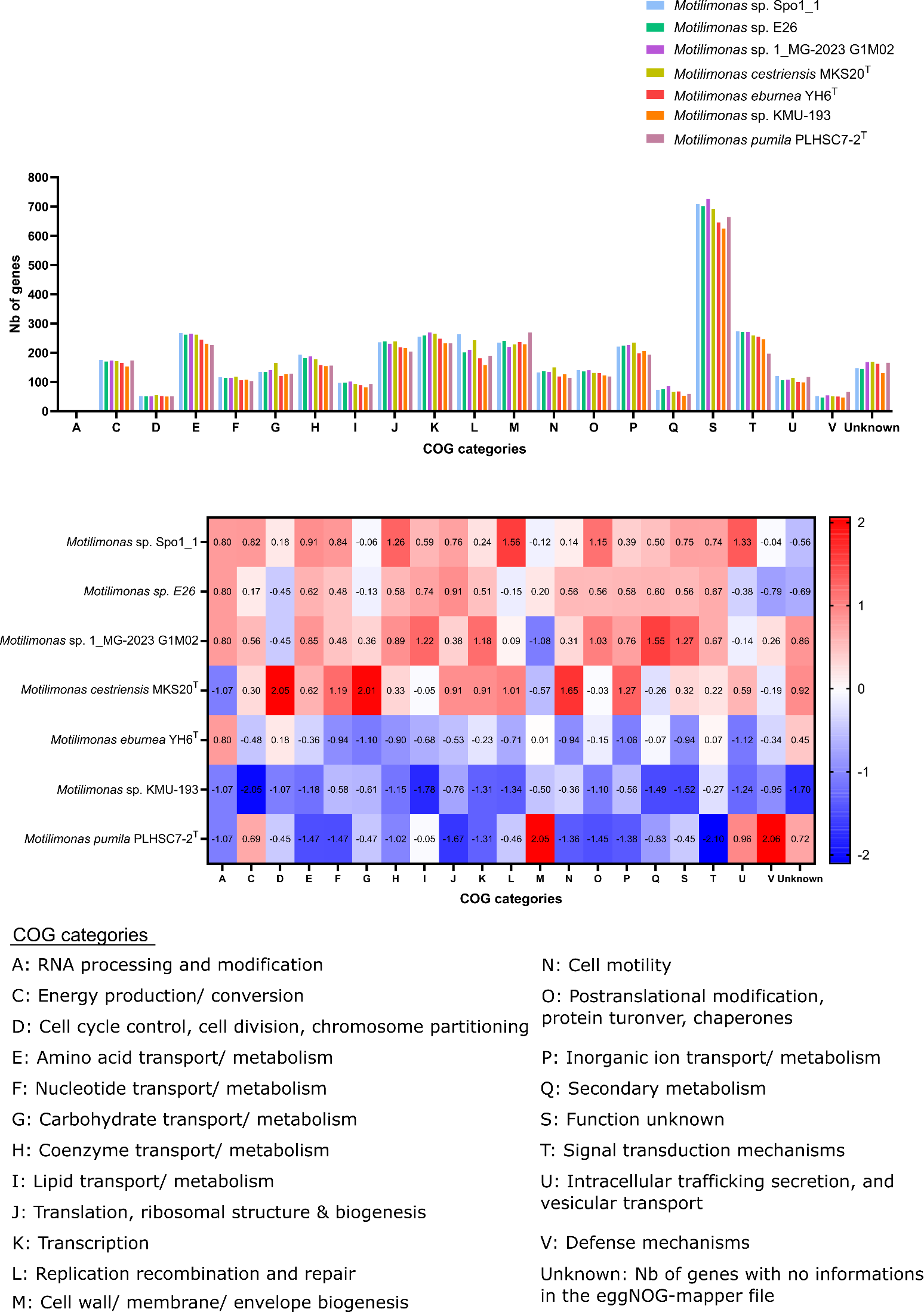


Figure S1: a) Functional classification of the different *Motilimonas* genomes based on COG assignments. b) Enrichment heatmap of COG categories in the *Motilimonas* genomes.
